# Extracellular Vesicles Derived From Plasma of Patients With Neurodegenerative Disease Have Common Transcriptomic Profiling

**DOI:** 10.3389/fnagi.2022.785741

**Published:** 2022-02-16

**Authors:** Daisy Sproviero, Stella Gagliardi, Susanna Zucca, Maddalena Arigoni, Marta Giannini, Maria Garofalo, Valentina Fantini, Orietta Pansarasa, Micol Avenali, Matteo Cotta Ramusino, Luca Diamanti, Brigida Minafra, Giulia Perini, Roberta Zangaglia, Alfredo Costa, Mauro Ceroni, Raffaele A. Calogero, Cristina Cereda

**Affiliations:** ^1^Genomic and Post-genomic Unit, Istituto di Ricovero e Cura a Carattere Scientifico (IRCCS) Mondino Foundation, Pavia, Italy; ^2^EnGenome SRL, Pavia, Italy; ^3^Department of Molecular Biotechnology and Health Sciences, Bioinformatics and Genomics Unit, University of Turin, Turin, Italy; ^4^Department of Brain and Behavioral Sciences, University of Pavia, Pavia, Italy; ^5^Department of Biology and Biotechnology (“L. Spallanzani”), University of Pavia, Pavia, Italy; ^6^Laboratory of Neurobiology and Neurogenetic, Golgi-Cenci Foundation, Milan, Italy; ^7^Neurorehabilitation Unit, IRCCS Mondino Foundation, Pavia, Italy; ^8^Unit of Behavioral Neurology, Istituto di Ricovero e Cura a Carattere Scientifico (IRCCS) Mondino Foundation, Pavia, Italy; ^9^Neuro-Oncology Unit, Istituto di Ricovero e Cura a Carattere Scientifico (SRCCS) Mondino Foundation, Pavia, Italy; ^10^Parkinson Disease and Movement Disorders Unit, Istituto di Ricovero e Cura a Carattere Scientifico (IRCCS) Mondino Foundation, Pavia, Italy

**Keywords:** neurodegenerative diseases, extracellular vesicles, RNA-Seq, mRNA, lncRNA

## Abstract

**Objectives:**

There is a lack of effective biomarkers for neurodegenerative diseases (NDs) such as Alzheimer's disease (AD), Parkinson's disease (PD), amyotrophic lateral sclerosis (ALS), and frontotemporal dementia. Extracellular vesicle (EV) RNA cargo can have an interesting potential as a non-invasive biomarker for NDs. However, the knowledge about the abundance of EV-mRNAs and their contribution to neurodegeneration is not clear.

**Methods:**

Large and small EVs (LEVs and SEVs) were isolated from plasma of patients and healthy volunteers (control, CTR) by differential centrifugation and filtration, and RNA was extracted. Whole transcriptome was carried out using next generation sequencing (NGS).

**Results:**

Coding RNA (i.e., mRNA) but not long non-coding RNAs (lncRNAs) in SEVs and LEVs of patients with ALS could be distinguished from healthy CTRs and from other NDs using the principal component analysis (PCA). Some mRNAs were found in commonly deregulated between SEVs of patients with ALS and frontotemporal dementia (FTD), and they were classified in mRNA processing and splicing pathways. In LEVs, instead, one mRNA and one antisense RNA (i.e., MAP3K7CL and AP003068.3) were found to be in common among ALS, FTD, and PD. No deregulated mRNAs were found in EVs of patients with AD.

**Conclusion:**

Different RNA regulation occurs in LEVs and SEVs of NDs. mRNAs and lncRNAs are present in plasma-derived EVs of NDs, and there are common and specific transcripts that characterize LEVs and SEVs from the NDs considered in this study.

## Introduction

Disease-specific mechanisms for neurodegenerative diseases (NDs) like Alzheimer's disease (AD), Parkinson's disease (PD), amyotrophic lateral sclerosis (ALS), and frontotemporal dementia (FTD) are more commonly investigated for specific therapeutic and diagnostic targets (Habib, 2018). NDs have a complex multifactorial nature, and preventive interventions that simultaneously target multiple risk factors and disease mechanisms at an early stage of the diseases might be effective. Therefore, studying the shared mechanisms and common biomarkers can facilitate the development of a better understanding in the prevention of these diseases. In this context, extracellular vesicles [EVs; large EVs (LEVs) and small EVs (SEVs)] are considered intriguing biomarkers. LEVs are vesicles of 100–1,000 nm, shed by budding of the plasma membrane of cells in homeostasis but particularly during pathological conditions, whereas SEVs are vesicles of 30–150 nm, which are formed intracellularly and released by exocytosis of multivesicular bodies (Cocucci and Meldolesi, 2015; Théry et al., 2018). EVs are produced by all types of cells, and they can help the shuttling of proteins, microRNAs (miRNAs), and mRNA in the intercellular space and in different body fluids. There are several studies on the role of EV cargo in cerebrospinal fluid (CSF) samples of patients with NDs (Burgos et al., 2014; Gui et al., 2015; Thompson et al., 2020). However, due to the invasiveness and risk on CSF collection, plasma-derived EVs are being studied. For instance, it has been proved that EVs are potential carriers of misfolded toxic proteins, such as amyloid-beta (Aβ) peptide and tau in AD (Saman et al., 2012; Rajendran et al., 2014; Ghidoni et al., 2018), α-synuclein in PD (Stuendl et al., 2021), and TDP-43 in ALS and FTD (Iguchi et al., 2016; Sproviero et al., 2018, 2019). Increased levels of t-tau, p-tau, and Aβ42 in plasma/serum neurally derived blood exosomes were demonstrated to be an early biomarker for AD and cognitive decline progression (Fiandaca et al., 2015). Moreover, several synaptic proteins were found reduced in blood-derived exosomes from patients with AD and FTD (Goetzl et al., 2015, 2018). Large EVs of patients with ALS carry more SOD1, TDP-43, and FUS compared with those of healthy control (CTR) (Sproviero et al., 2018). It has been reported that EVs are also loaded with different kinds of RNAs involved in intercellular communication. In general, EVs can transport protein coding RNA (i.e., mRNA) and non-coding species of RNA like miRNAs, t-RNA, rRNA, long non-coding RNAs (lncRNA), and piwi-interacting RNA (piRNA) (O'Brien et al., 2020). It is known that RNA metabolism is a common factor in the pathogenesis of NDs, and numerous new studies have been investigating the role of miRNAs in EVs of NDs (Liu et al., 2017; Jiang et al., 2019; Soares Martins et al., 2021). On this subject, we have recently demonstrated that plasma-derived SEVs and LEVs from AD, PD, ALS, and FTD carry different miRNAs and have a common signature among the four NDs (Sproviero et al., 2021). However, there is no evidence in the literature of the transcriptomic analysis of mRNAs and lncRNAs in EVs from NDs. The aim of this study was to fill this gap and describe mRNA and lncRNA involvement in LEVs and SEVs from plasma of patients with NDs.

## Materials and Methods

### Study Subjects

Patients were all recruited at the IRCCS Mondino Foundation, Pavia (Italy), and they signed an informed consent form approved by the Ethical Committee (for patients with ALS, Protocol *n*-20180034329; for patients with PD, Protocol n-20170001758; for patients with AD, Protocol *n*-20170016071; and for patients with FTD, Protocol *n*-20180049077). Six patients with AD, 9 patients with PD, 6 patients with sporadic ALS (SALS), and 9 patients with FTD were involved (Table 1). All patients were screened for mutations using next generation sequencing (Sure Select QXT Target Enrichment, Agilent Technology), and they did not carry genetic mutations. Diagnosis of AD was based on criteria expressed by Aging-Alzheimer's Association workgroups (McKhann et al., 2011). Extensive neuropsychological evaluation, 3T-MRI, and a 3-year follow-up were performed. Clinical diagnostic criteria of the Movement Disorder Society (MDS) were used for patients with PD; bradykinesia plus rest tremor or rigidity were taken in account. Rascovsky criteria were followed for patients with FTD (Rascovsky et al., 2011; Postuma et al., 2015). Only patients fulfilling clinical criteria for behavioral variant FTD (bv-FTD) or motor neuron disease (MND) were selected. ALS diagnosis was made according to the revised El Escorial Criteria (Brooks et al., 2000). Further clinical characteristics are listed in Tables 3–7 of the previous publication (Sproviero et al., 2021).

**Table 1:** Baseline characteristics of recruited subjects for this study.

	**CTRs**	**AD**	**FTD**	**ALS**	**PD**
Recruited subjects	6	6	9	6	9
Age (mean ± SD)	55 ± 5.2	77 ± 3.7	60 ± 6.7	72 ± 6.3	69 ± 3,6
Males %	43%	50%	78%	50%	60%
Females %	67%	50%	22%	50%	40%

*CTRs, controls; AD, Alzheimer's disease; FTD, fronto-temporal dementia; ALS, amyotrophic lateral sclerosis; PD, Parkinson's disease; SD, standard deviation. Age is reported as mean ± SD. The percentage of male and female subjects is also indicated*.

Six age-matched healthy volunteers, free from any pharmacological treatment, were recruited and used as healthy CTRs. CTRs were recruited at the Immunohematological and Transfusional Service, IRCCS Foundation “San Matteo,” Pavia (Italy).

### Isolation of LEVs and SEVs

Venous blood (15 ml) was collected in sodium citrate tubes from all patients and CTRs and processed as previously described (Sproviero et al., 2018, 2019, 2021; Morasso et al., 2020). In brief, platelet-free plasma was centrifuged at 20,000 × *g* for 1 h. The pellet was washed in 0.2 μm filter and filtered with 1 × PBS (Sigma-Aldrich, Italy). The supernatant of LEVs was filtered through a 0.2 μm filter and spun in an Optima MAX-TL Ultracentrifuge at 100,000 × *g* for 1 h at 4°C, and the SEV pellet was washed with 1 ml of filtered 1 × PBS. The purity of LEVs and SEVs was confirmed by the Western blot (WB) analysis **(**using anti-Annexin V antibody-Santa Cruz Biotechnology, USA, for LEVs and anti-Alix antibody-Abcam, Cambridge, UK, for SEVs), nanotracking particle analysis (NTA), and transmission electron microscopy (TEM) as we described previously (Sproviero et al., 2021).

### RNA Extraction

RNA was extracted from LEV and SEV fractions using the Qiagen miRNeasy Mini kit (Qiagen, Germany), according to the instructions of the manufacturer. RNA was quantified using a Nanodrop ND-100 Spectrophotometer (Nanodrop Technologies, Wilmington, USA) and a 2100 Bioanalyzer (Agilent RNA 6000 Nano Kit, Waldbronn, Germany). The RNA quantity used was ~200ng.

### RNA Libraries Preparations

Long RNA libraries (i.e., mRNAs and lncRNAs) were prepared using the Illumina TruSeq Stranded RNA Library Prep (Illumina, USA). Sequencing (i.e., nts paired-end) was performed on the Illumina NextSeq500 system (Illumina, USA).

### Bioinformatics Analysis

The raw bcl files were converted into demultiplexed fastq files with bcl2fastq (Illumina, USA) implemented in the docker4seq package (Kulkarni et al., 2018). For the row count analysis, only transcripts with counts above five were considered (see Supplementary Figure 1). No relevant difference among counts in SEVs and LEVs in the four diseases emerged. The gene and isoform quantification was performed as previously described (Gagliardi et al., 2018; Zucca et al., 2019). The differential expression analysis for mRNAs was performed using the R package EBSeq (Langfelder and Horvath, 2008). Coding and non-coding genes were considered differentially expressed and retained for further analysis with |log2(disease sample/healthy control)| ≥ 1 and an FDR ≤ 0.1. We imposed minimum |Log2FC| of 1 and an FDR lower than 0.1 as thresholds to differentially expressed genes (DEGs). To understand common RNAs in the four diseases, we calculated the intersection of deregulated mRNAs and lncRNAs compared with CTRs with http://bioinformatics.psb.ugent.be/webtools/Venn/. The datasets generated and analyzed during this study are available in the NCBI GEO repository [GSE155700].

### Pathways Analysis

The gene enrichment analysis was performed on coding genes with the KEGG pathway analysis (Kyoto Encyclopedia of Genes and Genomes, http://www.genome.ad.jp/kegg) and enrichR web tool (Chen et al., 2013; Kuleshov et al., 2016). Only pathways with *p* < 0.05 are considered statistically significant.

### Statistical Analysis

The statistical analysis was carried out using the R Studio software. The Shapiro-Wilk test was used to test variables for normality distribution, and the Levene test was used to test the assumption of homogeneity. Categorical variables were tested using the non-parametric Kruskal-Wallis with Dunn's multiple comparison test, and a value of *p* < 0.05 was considered significant.

## Results

### Number and Selective mRNAs and lncRNAs in SEVs and LEVs

Large extracellular vesicles (LEVs) and SEVs were separated by differential centrifugation. NTA, WB analysis, and TEM were carried out (Supplementary Figure 2) to establish the purity of EVs. The concentration and diameter size of LEVs and SEVs of CTRs and patients are shown in Supplementary Figure 3. The diameter of LEVs in patients with ALS is enhanced compared with that of CTRs (Dunn's test, *p* < 0.001; Supplementary Figure 3A). The diameter of SEVs among the four NDs had a slight difference (Supplementary Figure 3C). Only patients with PD showed more LEVs compared with CTRs and other groups (^*^p < 0.05; Supplementary Figure 3B). The number of SEVs of ALS and FTD was enhanced compared with that in CTRs (i.e., Dunn's test, ^**^p < 0.01, and ^****^p < 0.001; Supplementary Figure 3D). These data have to be confirmed with more patients and CTRs.

Differentially expressed mRNA (DE mRNA) and DE lncRNAs between LEVs and SEVs of the 4 NDs (i.e., AD, PD, ALS, and FTD) and the healthy CTRs were calculated, and the results are shown in Table 2 and Supplementary Table 1. SEVs from patients with ALS and FTD were more enriched in DE mRNAs compared with those in LEVs. As shown in Table 2, LEVs and SEVs from patients with ALS and FTD were equally enriched in lncRNAs. However, no DE mRNAs and lncRNAs were found in plasma-derived EVs from patients with AD, and only 12 DE mRNAs and 1 lncRNA were found in LEVs from plasma of patients with PD.

**Table 2:** Statistically significant differentially expressed RNAs number in small extracellular vesicles (SEVs) and large extracellular vesicles (LEVs) from patients with Alzheimer's disease (AD), frontotemporal dementia (FTD), amyotrophic lateral sclerosis (ALS), and Parkinson's disease (PD) in terms of upregulated transcripts, downregulated transcripts, and total compared with CTRs. Transcripts were considered as differentially expressed when |log2(disease sample/healthy control)|≥1 and an FDR ≤ 0.1.

	**AD**		**FTD**		**ALS**		**PD**	
	***SEVs***	***LEVs***	***SEVs***	***LEVs***	***SEVs***	***LEVs***	***SEVs***	***LEVs***
mRNA up-regulated	0	0	194	64	499	32	0	2
mRNA down-regulated	0	0	33	23	43	56	0	10
Total	0	0	227	87	542	88	0	12
lncRNA up-regulated	0	0	9	9	12	4	0	0
lncRNA down-regulated	0	0	0	2	0	15	0	1
Total	0	0	9	11	12	19	0	1

*AD, Alzheimer's disease; FTD, fronto-temporal dementia; ALS, amyotrophic lateral sclerosis; PD, Parkinson's disease; CTRs, controls; SEVs, small extracellular vesicles; LEVs, large extracellular vesicles; lncRNAs, long non-coding RNAs; FDR, false discovery rate*.

**Figure 1:**
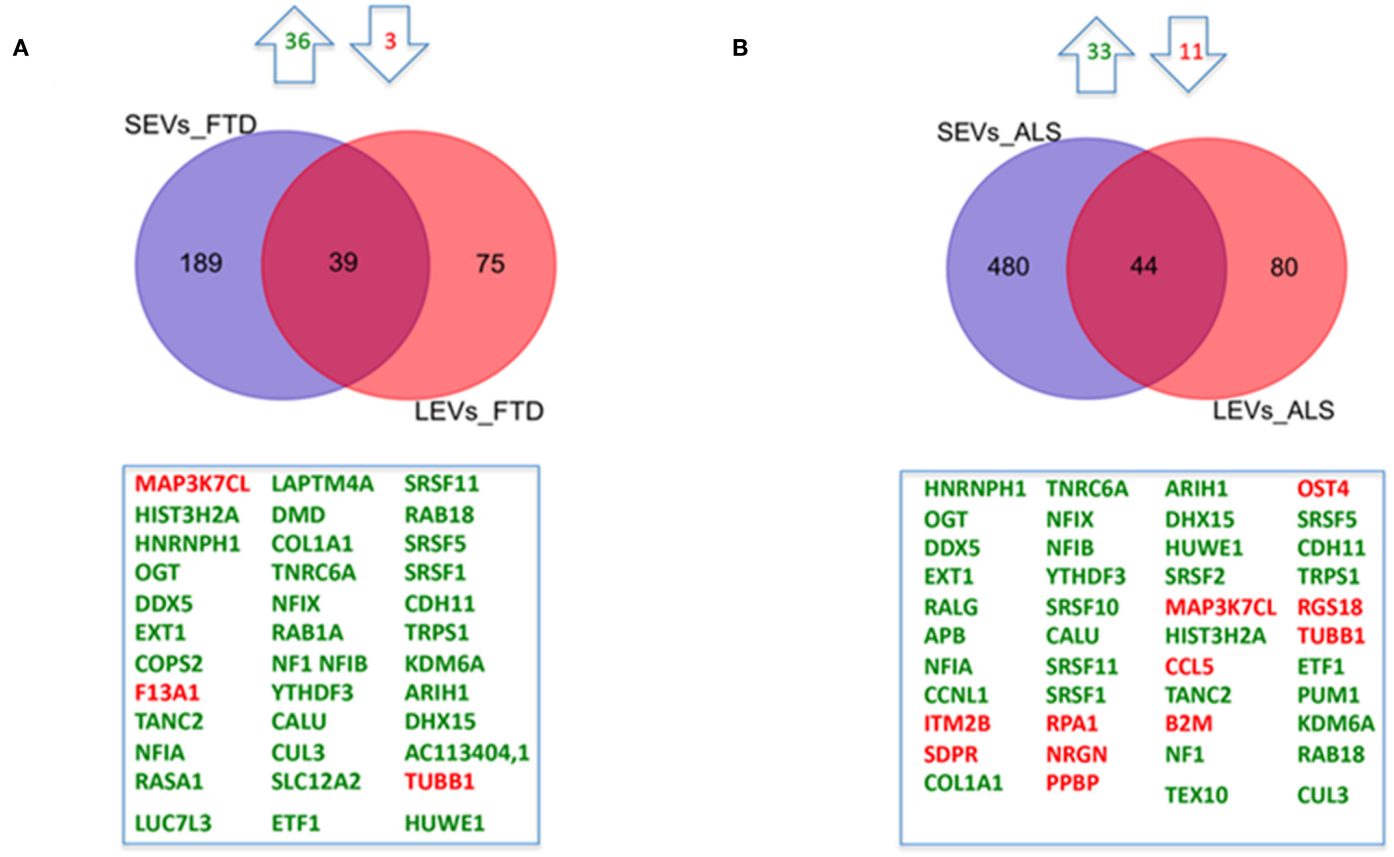
Common packaging of deregulated mRNAs into small extracellular vesicles (SEVs) and large extracellular vesicles (LEVs) from patients with frontotemporal dementia (FTD) and amyotrophic lateral sclerosis (ALS). **(A)** For FTD, of the 228 mRNA in SEVs and 114 in LEVs, 39 were in common (i.e., 36 upregulated, green, and 3 downregulated, red). **(B)** For ALS, of the 522 mRNA in SEVs and 124 in LEVs, 44 were in common (i.e., 33 upregulated and 11 downregulated). Differential mRNA expression analysis was carried out using DESeq2 (log2FC > 1, *p* < 0.05).

**Figure 2:**
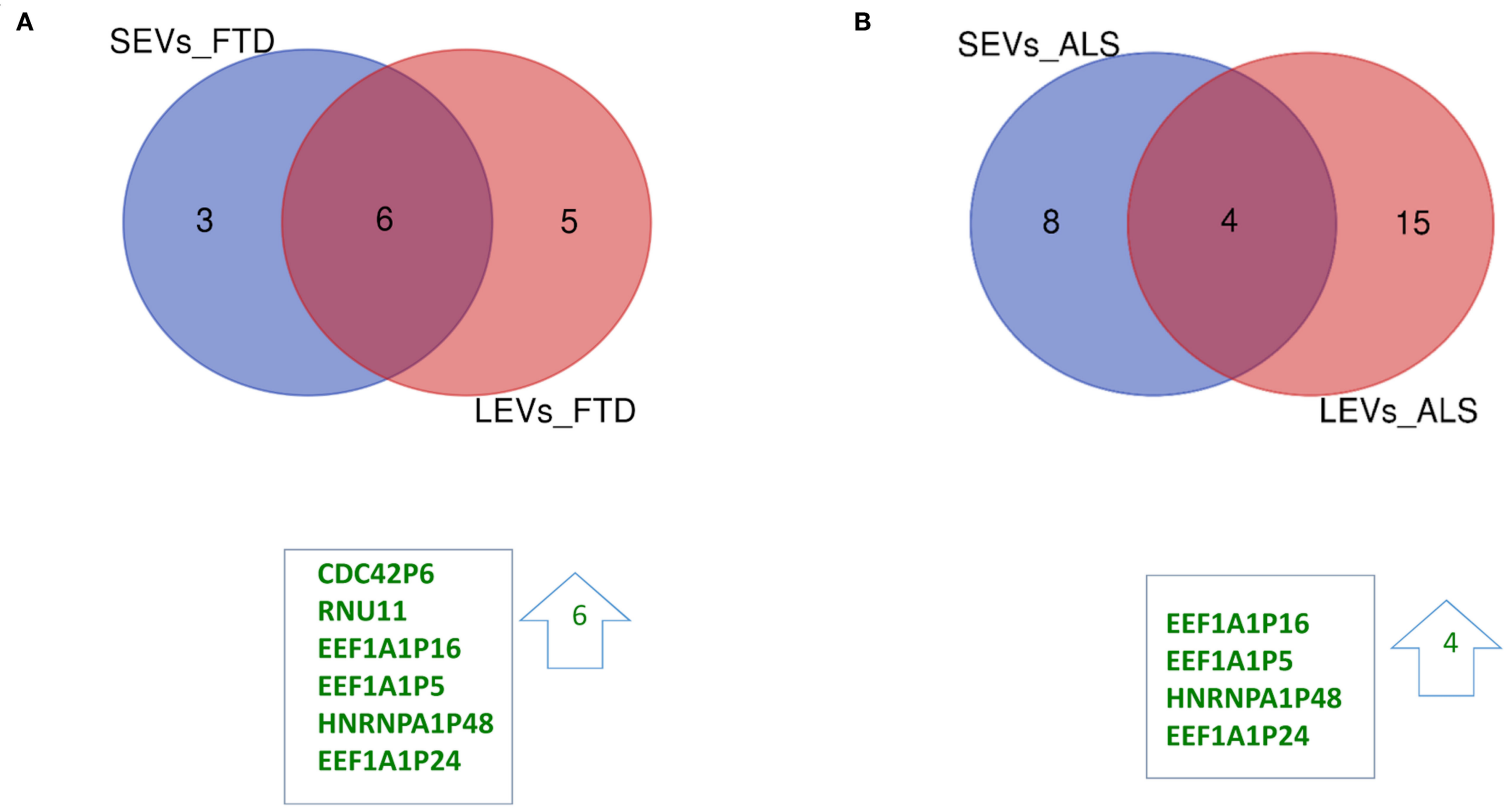
Common packaging of deregulated long non-coding RNAs (lncRNAs) and pseudogenes into SEVs and LEVs from FTD and ALS patients. **(A)** For FTD, of the 9 lncRNAs in SEVs and 11 in LEVs, 6 were in common (all upregulated, green). **(B)** For ALS, of the 12 lncRNA in SEVs and 19 in LEVs, 4 were in common (all upregulated, green). Differential lncRNA expression analysis was carried out using DESeq2 (log2FC> 1, *p* < 0.05).

We have largely demonstrated significant differences between LEVs and SEVs derived from plasma for dimension, markers, protein, and miRNA loading (Sproviero et al., 2018, 2019, 2021). As reported in our previous work (Sproviero et al., 2021), miRNAs can sort differently in LEVs and SEVs of the same disease. So, we moved to investigate the number of different and common deregulated mRNAs and lncRNAs that sort into SEVs and LEVs in the same disease. We could only calculate the intersection between SEVs and LEVs for ALS and FTD, since there were no deregulated mRNAs (with a Log2FC| of 1) in EVs of patients with AD and in SEVs of patients with PD compared with those in CTRs. For FTD, of the 228 mRNAs in SEVs and 114 in LEVs, 39 mRNAs were in common, 36 were upregulated, and 3 were downregulated (Figure 1A). For ALS, of the 522 mRNA in SEVs and 124 in LEVs, 44 were in common (i.e., 33 upregulated and 11 downregulated; Figure 1B). The percentage of common RNAs between SEVs and LEVs are shown in Table 3. For FTD, of the 9 lncRNA in SEVs and 11 in LEVs, 6 were in common (Figure 2A). For ALS, of the 12 lncRNA in SEVs and 19 in LEVs, 4 were in common (Figure 2B).

**Table 3:** Percentage of common mRNAs and long non-coding RNAs (lncRNAs) in SEVs and LEVs in AD, FTD, ALS, and PD.

**mRNAs**		
	**Common mRNAs in SEVs and LEVs (%)**	
	***SEVs***	***LEVs***
AD	0	0
FTD	17.1	34.2
ALS	8.4	35.5
PD	0	0
**lncRNAs**		
	**Common lncRNAs in SEVs and LEVs (%)**	
	***SEVs***	***LEVs***
AD	0	0
FTD	33.3	21.1
ALS	66.7	54.5
PD	0	0

*AD, Alzheimer's disease; FTD, fronto-temporal dementia; ALS, amyotrophic lateral sclerosis; PD, Parkinson's disease; CTRs, controls; SEVs, small extracellular vesicles; LEVs, large extracellular vesicles; mRNAs, messangerRNAs; lncRNAs, long non-coding RNA*.

### RNA Expression Profiles and Common Pathways in SEVs and in LEVs of NDs

**Figure 3:**
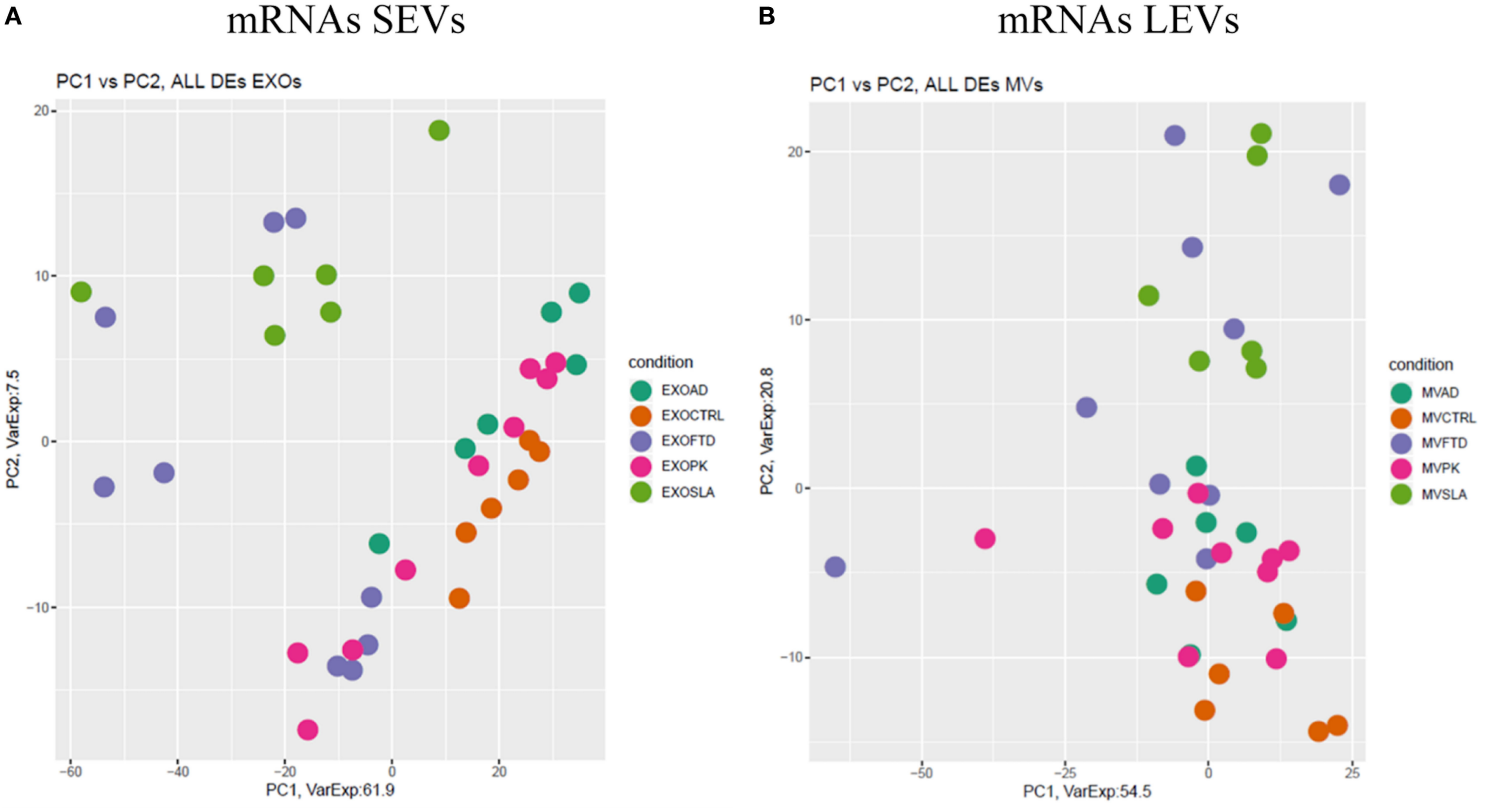
Principal component analysis (PCA) of coding genes differentially expressed in SEVs **(A)** and LEVs **(B)** of patients with AD, FTD, ALS, and PD and healthy controls (CTRs). PCA is performed using all the differentially expressed coding genes in at least one disease as predictors in the comparison of each disease to the control state. Each dot represents a sample, and each color represents a disease.

**Figure 4:**
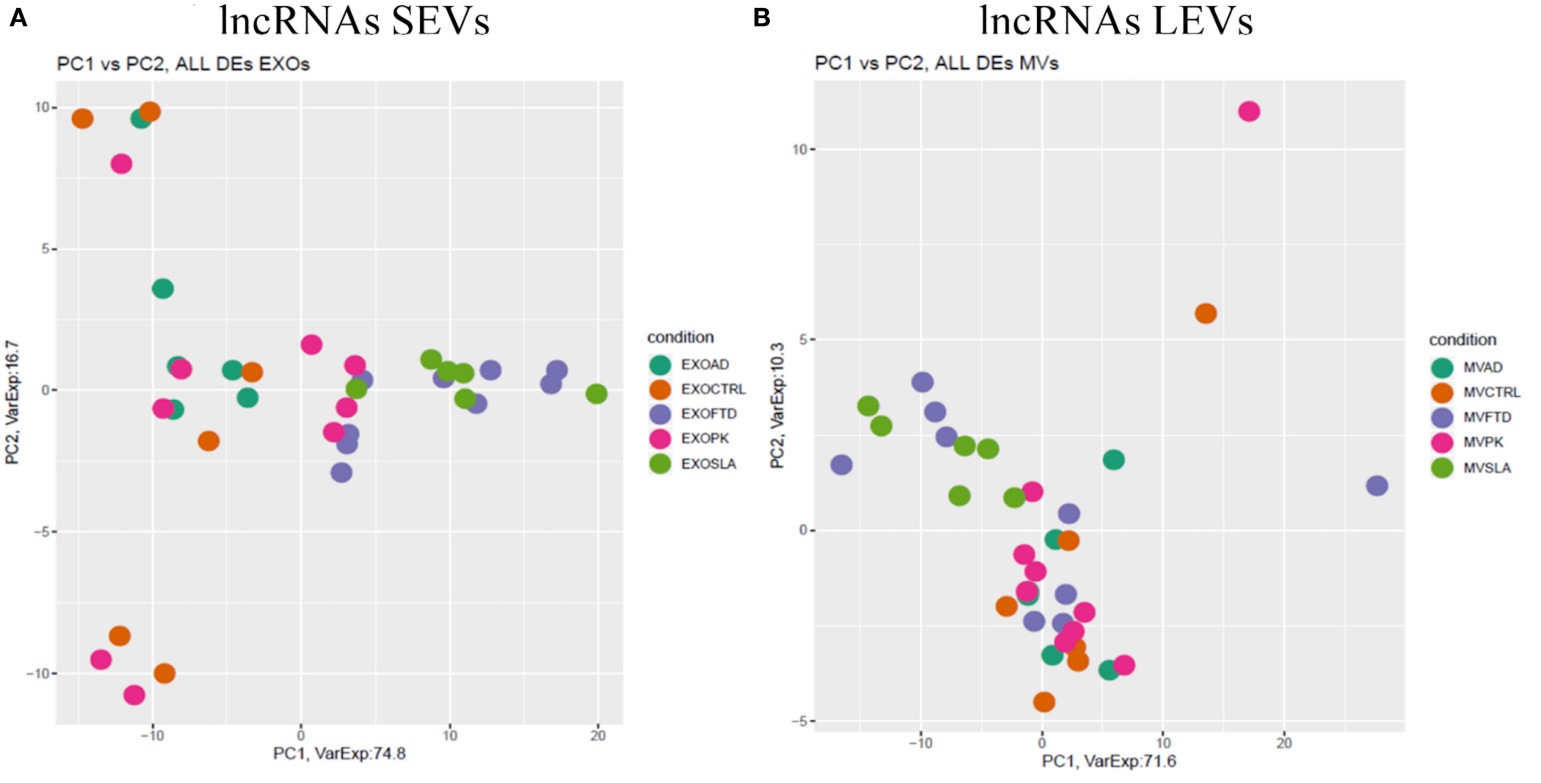
PCA of long non-coding genes differentially expressed in SEVs **(A)** and LEVs **(B)** of patients with AD, FTD, ALS, and PD and healthy CTRs. PCA is performed using all DE long non-coding genes in at least one disease as predictors in the comparison of each disease to the control state. Each dot represents a sample, and each color represents a disease. Considering only lncRNAs, both SEVs and LEVs showed a mixed scenario between patients and controls, without any specific characterization of AD, FTD, ALS, or PD.

**Figure 5:**
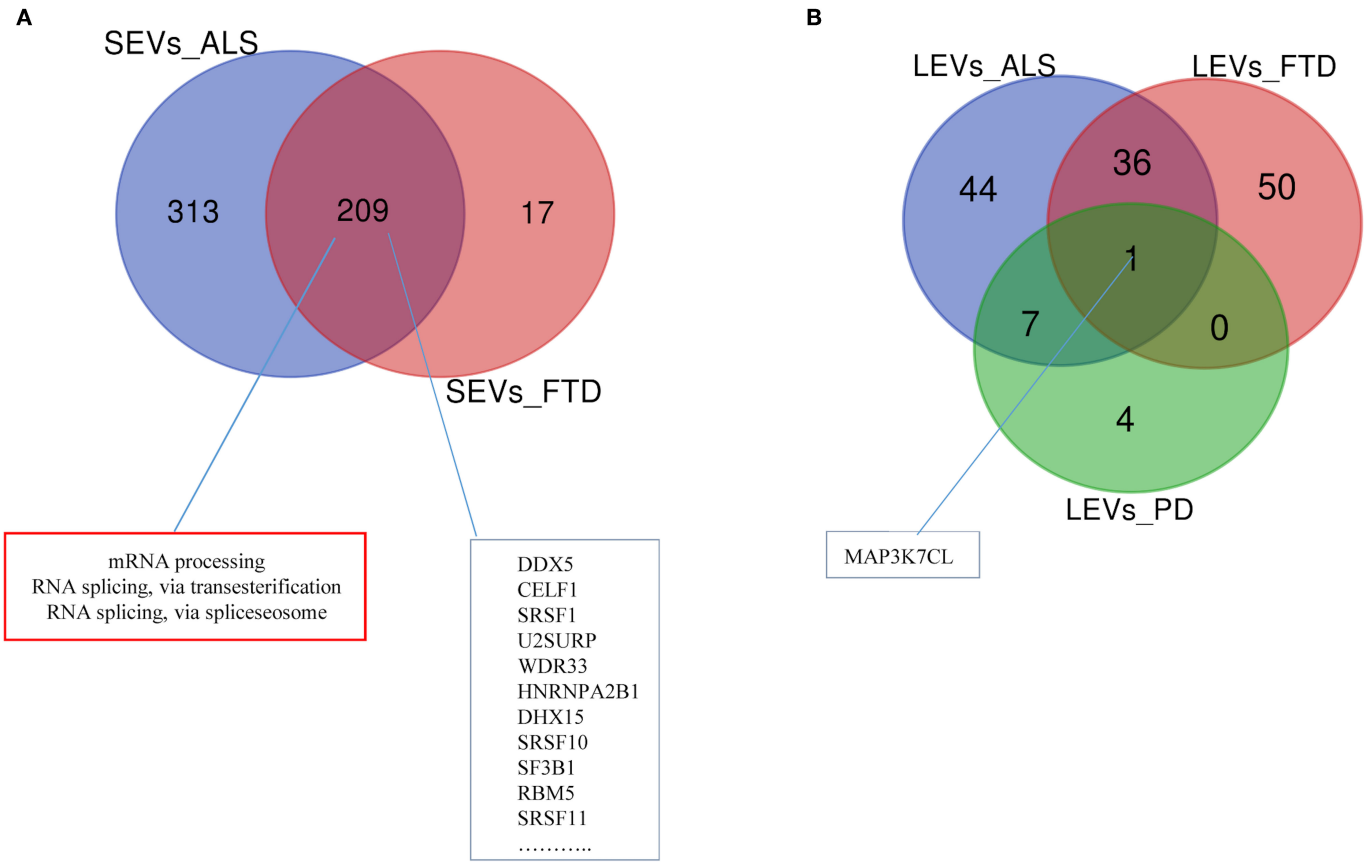
Venn diagram showing numbers of common and unique RNA in SEVs from plasma of patients with ALS and FTD **(A)** and in LEVs from plasma of ALS, FTD, and PD **(B)**. Common mRNAs and pathways are listed. Differential mRNA expression analysis was carried out using DESeq2 (log2FC> 1, *p* < 0.05).

**Figure 6:**
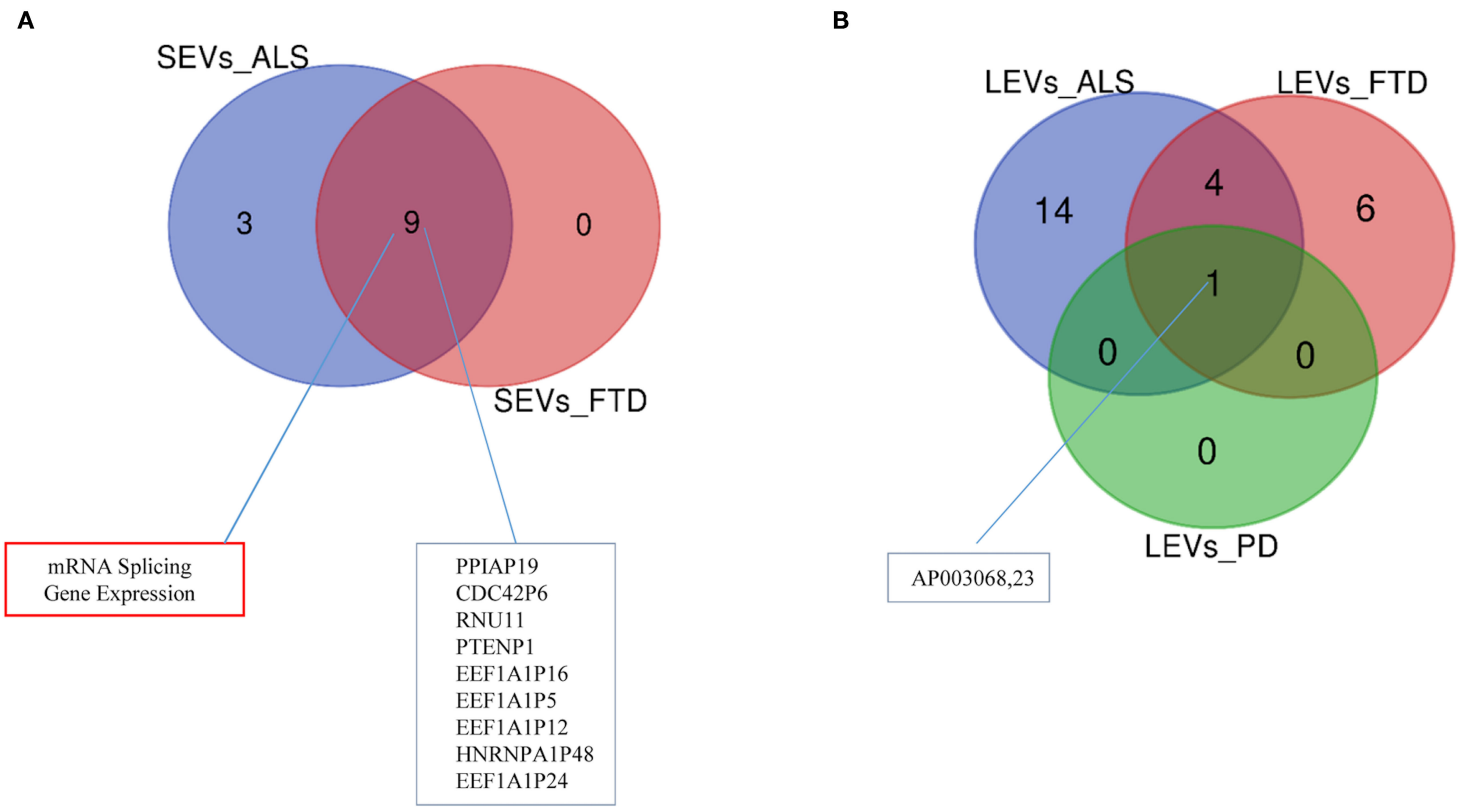
Venn diagram showing numbers of common and unique lncRNAs and pseudogenes in SEVs from plasma of patients with ALS and FTD **(A)** and in LEVs **(B)** from plasma of ALS, FTD, and PD. Common lncRNAs and pathways are listed. Differential mRNA expression analysis was carried out using DESeq2 (log2FC > 1, *p* < 0.05).

Differentially expressed mRNAs and lncRNAs in SEVs and LEVs of the four groups (Supplementary Table 1) were analyzed, and the principal component analysis (PCA) was carried out. DE mRNAs and lncRNAs in SEVs and LEVs showed that patients with ALS are well-divided from CTRs (Figures 3, 4), showing a partial overlap with FTD. Common DE mRNAs and lncRNAs between LEVs or SEVs of the four diseases were investigated. In SEVs from patients with FTD and ALS, there were 209 mRNA and 9 lncRNAs in common, and the common pathways of gene ontology biological processes (GO-BPs) were mRNA processing (*p* = 6.133e-15), RNA splicing, *via* transesterification reactions, and *via* spliceosome (Figures 5A, 6A). In LEVs, there was only 1 mRNA MAP3K7CL, a kinase gene, and one antisense, AP003068.3, which are in common among PD, FTD, and ALS (Figures 5B, 6B).

### Pathways Analysis of mRNAs and lncRNAs in SEVs and LEVs

The EnrichR analysis (i.e., KEGG pathway and Gene Ontology analysis) for DE mRNA in patients with FTD and ALS compared with that in healthy CTRs has been performed (Supplementary Table 2). GO-BPs identified (1) positive regulation of transmembrane transport and of leukocyte chemotaxis in LEVs from patients with ALS; (2) cellular response to molecule of bacterial origin in LEVs from patients with FTD; (3) positive regulation of mitochondrial membrane permeability involved in apoptotic process and mitochondrial outer membrane permeabilization in LEVs from patients with PD; (4) regulation of transcription from an RNA polymerase II promoter, regulation of transcription, DNA-templated, and negative regulation of gene expression in SEVs from ALS; (5) regulation of cellular catabolic process, phosphatidylinositol metabolic process, and transcription initiation from RNA polymerase III promoted in SEVs from patients with FTD (Supplementary Table 2).

## Discussion

As widely reported in the literature, EVs can transport full-length and fragmented RNAs (Skog et al., 2008). Protein-coding RNAs are synthesized in the nucleus; they undergo splicing, are modified at the ends, and are exported to the cytosol, where they can be released by EVs from the cell. The transcriptomic analysis supported the view that mRNA from the parent cell can be translated in the recipient cell to generate a functional protein. For example, it was found that 17% of the total mRNAs found in EVs were not detected in parental cells, and more than 11% of mRNAs were preferentially included in EVs compared with mRNAs found in cells (Skog et al., 2008).

Long non-coding RNAs, defined as >200 nt transcripts that are not translated into proteins, are involved in different biological processes such as chromatin organization, gene transcription, mRNA turnover, protein translation, and assembly of macromolecular complexes. Numerous lncRNAs have been found in EVs. For example, MALAT1, HOTAIR, lincRNAp21, GAS5, TUG1, and ncRNA-CCND1 were identified in EVs derived from human cervical and breast carcinomas (Gezer et al., 2014).

In this study, we have analyzed SEV and LEV cargo to detect RNAs acting as novel, easily accessible biomarkers for AD, PD, ALS, and FTD. We first compared DE mRNAs and lncRNAs between SEVs and LEVs in the same disease. We only found a partial overlap between SEVs and LEVs for mRNAs (Table 3). We could not evaluate the overlap between DE mRNAs of SEVs and LEVs of AD and PD, since we did not find any DE mRNAs in patients with AD and only few in LEVs of patients with PD. Although there is some overlap between the two types of EVs, there is a significant difference. This could be justified by the different functions of LEVs and SEVs in plasma of patients with ND, as we previously described for dimension, protein, miRNAs, and lipid loading (Sproviero et al., 2018, 2019, 2021). In diseases like FTD and ALS, SEVs were enriched with a greater number of DE mRNAs compared with LEVs. FTD and ALS are linked clinically, pathologically, and mechanistically. Mutations in TDP-43 and FUS/TLS were discovered to be common in both diseases. Moreover, TDP-43 was found to be the major ubiquitinated protein found in both patients with ALS and FTD (Arai et al., 2006; Neumann et al., 2006). These findings highlighted the dysfunctions in RNA metabolism and multiple RNA processing steps as central pathogenic pathways in ALS and FTD (Kwiatkowski et al., 2009; Lagier-Tourenne et al., 2010). However, many ALS/FTD-linked proteins are involved in the autophagy pathways (like ubiquilin-2-UBQLN2-, p62/SQSTM1, and optineurin-OPTN-) and in the endosomal sorting complex (like CHMP2B, a core component of endosomal sorting complex required for transport (ESCRT) complexes) (Ling et al., 2013). Being the exosomal pathway linked to autophagy and to the endolysosomal pathway (Buratta et al., 2020), this might justify the enhanced deregulation of RNA observed in SEVs.

We then analyzed DE mRNAs among the four NDs by the PCA. We found that DE mRNA cargo from ALS and FTD was different from CTRs, particularly, in SEVs, while in LEVs, the only group that did not overlap with CTRs was ALS. PCA related to lncRNAs in LEVs and SEVs could only distinguish ALS from CTRs.

Common genes in SEVs of ALS and FTD were mainly classified as belonging to splicing, a mechanism largely described in the literature for these two NDs (Ito et al., 2017). For example, we found upregulation of serine arginine-rich protein family (SRs), including SRSF7, SRSF3, SRSF1, SRSF2, SRSF11, and SRSF10 in SEVs from patients with ALS and SRSF1, SRSF11, SRSF2, SRSF5, and SRSF10 in SEVs from patients with FTD. SRs are serine/arginine-rich splicing factors that are formed by an N-terminal 1 or 2 RNA recognition domains (RRM domain) and a C-terminal domain enriched with arginine (R) and serine (S) amino acid sequences (RS domain). SR proteins are involved in RNA metabolism and constitutive or alternative splicing, since they bind splicing enhancers [i.e., exonic splicing enhancer (ESE)] and recruit small nuclear RNP (snRNP) (Zheng et al., 2020).

Moreover, upregulation of HNRNPA2B1, Matrin3, and HNRNPA1P48 in SEVs of both patients with ALS and FTD, as well as TARDBP, identified ALS-FTD-related RNA-binding proteins, characteristically deposited in the affected regions of the brain of ALS/FTD (Ling et al., 2013).

One common gene, MAP3K7CL (a kinase gene), and one antisense, AP003068.3, were identified among LEVs of PD, ALS, and FTD. Not much is known about their role in neurodegeneration. Merienne and collaborators identified highly selective transcriptomic signatures in adult mouse striatal direct and indirect spiny projection neurons (SPNs), astrocytes, and microglia (Merienne et al., 2019). MAP3K7CL was one of the genes identified in SPNs of the striatonigral. The loss of striatal efferent neurons has been described in Huntington disease and X-linked recessive dystonia parkinsonism (Reiner et al., 1988; Goto et al., 2005); however, striatal efferent projections can be involved in the TDP-43-related FTLD/ALS disease (Riku et al., 2016).

We found 36 mRNAs and 4 non-coding RNAs in common between LEVs from ALS and FTD. mRNAs are involved in splicing and gene transcription. Even if common RNAs in SEVs and LEVs are both involved in splicing, only 15 mRNAs (i.e., CUL3, HIST3H2A, SRSF11, RAB18, SRSF1, DDX5, TRPS1, NFIA, ARIH1, DHX15, NFIX TUBB1, NF1, NFIB, and HUWE1) are shared, meaning that a specific common signature can be described for the two EV subpopulations.

One striking result from our RNA-Seq analysis is the small number of DE genes found in PD LEVs and the lack of DE genes and lncRNAs/pseudogenes in patients with AD and in SEVs from patients with PD compared with CTRLs. There are existing studies on plasma-derived EVs of patients with AD and their miRNA cargo analyzed with the high-throughput sequencing technology. Three studies confirmed the deregulation of miRNA-342-3p in plasma-derived exosomes from patients with AD (Tan et al., 2014; Lugli et al., 2015; Rani et al., 2017) compared with that in healthy donors. Downregulation of miRNA-21-5p, miRNA-451a (Gámez-Valero et al., 2019), and miRNA-132 and miRNA-212 (Cha et al., 2019) was found in exosomes of patients with AD compared with that in patients with DLB and healthy donors. Other works reported the presence of specific miRNAs in EVs from periphery of AD (Cheng et al., 2020; Thomas et al., 2020). We previously demonstrated a moderate presence of deregulated miRNAs in EVs from patients with PD and AD (Sproviero et al., 2021). Kim et al. (2020) reported a significant enrichment in mitochondrial RNAs in EVs isolated from blood of AD groups; however, there is no mention of DE coding and other non-coding RNAs (Kim et al., 2020).

Specific pathways are also described for each disease, and different DE genes are recognized in LEVs and SEVs of the same disease. Further work is needed to unravel specific signature for each disease.

Our previous work showed a different grade of RNA metabolism involvement in AD, ALS, and PD by investigating mRNAs and lncRNA in peripheral blood mononuclear cells (PBMC). Specifically, we found a great difference in the amount of DE mRNAs in the three diseases (Garofalo et al., 2020). These latter data are in line with the results described in this article, taking in account that patients with FTD were not included in the PBMC study. Further studies are needed on extended cohorts of patients with different severity of the disease to understand if the deregulation of these RNAs may be associated to a specific clinical window (i.e., disease onset and outcome) and progression of the disease.

This study may improve our understanding of mechanisms underlying neurodegeneration overlap, and it might help to develop diagnostics based on detection of RNAs in blood of patients.

## Data Availability Statement

The datasets presented in this study can be found in online repositories. The names of the repository/repositories and accession number(s) can be found in the article/Supplementary Material.

## Ethics Statement

The studies involving human participants were reviewed and approved by the Ethical Committee (for ALS patients Protocol n°-20180034329; for PD patients Protocol n°20170001758; for AD patients Protocol n°20170016071; and for FTD patients Protocol n°20180049077). The patients/participants provided their written informed consent to participate in this study.

## Author Contributions

DS, SG, and SZ wrote the manuscript. DS, MAr, MGi, MGa, and VF performed the experiments. RC and SZ performed bioinformatic analysis. AC, BM, GP, MAv, LD, RZ, MC, and MR participated in patients and controls recruitment. MAr, DS, OP, and CC set up the experimental plan. OP, RC, and CC reviewed the manuscript. RC and CC supervised this work. All authors reviewed and accepted the final version of this manuscript.

## Funding

This research was funded by Italian Ministry of Health (Grant N°5^*^1000 anno 2016, Ricerca Corrente 2018–2020, Young research project GR-2016-02361552, AIFA-co-ALS); EuroNanoMed III JTC 2018; Fondazione Regionale per la Ricerca Biomedica for TRANS–ALS (FRRB 2015-0023); and Fondazione Cariplo 2017 (Extracellular vesicles in the pathogenesis of Frontotemporal Dementia 2017-0747 and Association between frailty trajectories and biological markers of aging 2017-0557).

## Conflict of Interest

SZ was employed by EnGenome SRL. The remaining authors declare that the research was conducted in the absence of any commercial or financial relationships that could be construed as a potential conflict of interest.

## Publisher's Note

All claims expressed in this article are solely those of the authors and do not necessarily represent those of their affiliated organizations, or those of the publisher, the editors and the reviewers. Any product that may be evaluated in this article, or claim that may be made by its manufacturer, is not guaranteed or endorsed by the publisher.

## Supplementary Material

Supplementary Figure 1 Row counts for small and large extracellular vesicles. **(A)** Only transcripts with counts above five were considered. **(B)** No relevant difference between count in SEVs and LEVs in four diseases emerged.

Supplementary Figure 2 Large extracellular vesicles (LEVs) and small extracellular vesicles (SEVs) characterization. **(A)** Nanosight profile of LEVs and SEVs from plasma of a control (CTR); **(B)** Representative images obtained by transmission electron microscopy (TEM) of LEVs and SEVs from plasma (Scale bar: 100 nm). **(C)** Western blot of LEVs and SEVs markers in LEVs and SEVs samples from two CTRs showed the presence of Annexin V only in the LEV pellet and more Alix presence in SEV fraction.

Supplementary Figure 3 Mean diameter and particle count concentration for LEVs and SEVs of the four NDs and CTRs. NTA was performed on LEVs and SEVs extracted from plasma of healthy controls and neurodegenerative diseases (NDs). The mean diameter size and count of LEVs **(A,B)** and SEVs **(C,D)** of CTRs and patients are reported in the boxplots (Dunn's test, ^*^*p* < 0.05, ^**^*p* < 0.01, ^***^*p* < 0.001, and ^****^*p* < 0.0001) **(D)**. These data have to be confirmed with more patients and controls.

Supplementary Table 1 Differentially expressed RNAs in ALS, frontotemporal dementia (FTD), Parkinson's disease (PD), and Alzheimer's disease (AD) groups respect to healthy controls. Transcript ID, gene name, gene type, gene status, measured log2FC, and false discovery rate (FDR) are reported for each transcript. Only transcripts with | log2 (disease sample/healthy control)|≥1 and an FDR ≤ 0.1 are shown.

Supplementary Table 2 Specific mRNAs pathways in ALS, FTD, PD and AD groups. Deregulated mRNAs specific of each disease were analyzed using the EnrichR web tool. KEGG and Gene Ontology Biological Processes with a *p* < 0.05 are listed.
