# Supplementary figures and images for: Extracellular Vesicles Derived From Plasma of Patients With Neurodegenerative Disease Have Common Transcriptomic Profiling
Source: Front Aging Neurosci. 2022 Feb 16;14:785741. doi: 10.3389/fnagi.2022.785741 (PMC8889100; doi:10.3389/fnagi.2022.785741)

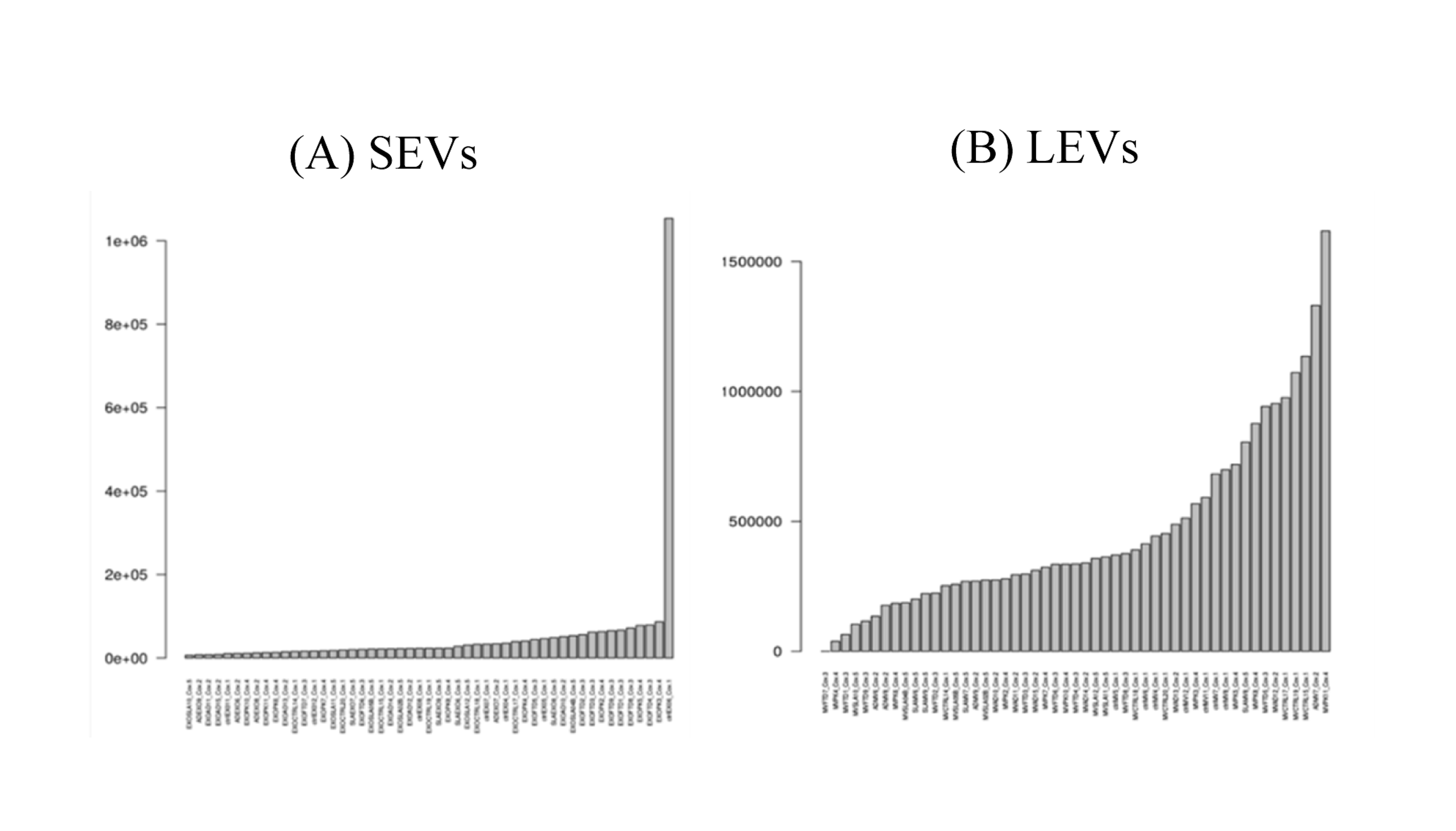

Supplement: Supplementary Figure 1 — Row counts for small and large extracellular vesicles. (A) Only transcripts with counts above five were considered. (B) No relevant difference between count in SEVs and LEVs in four diseases emerged. [file Image_1.TIF]

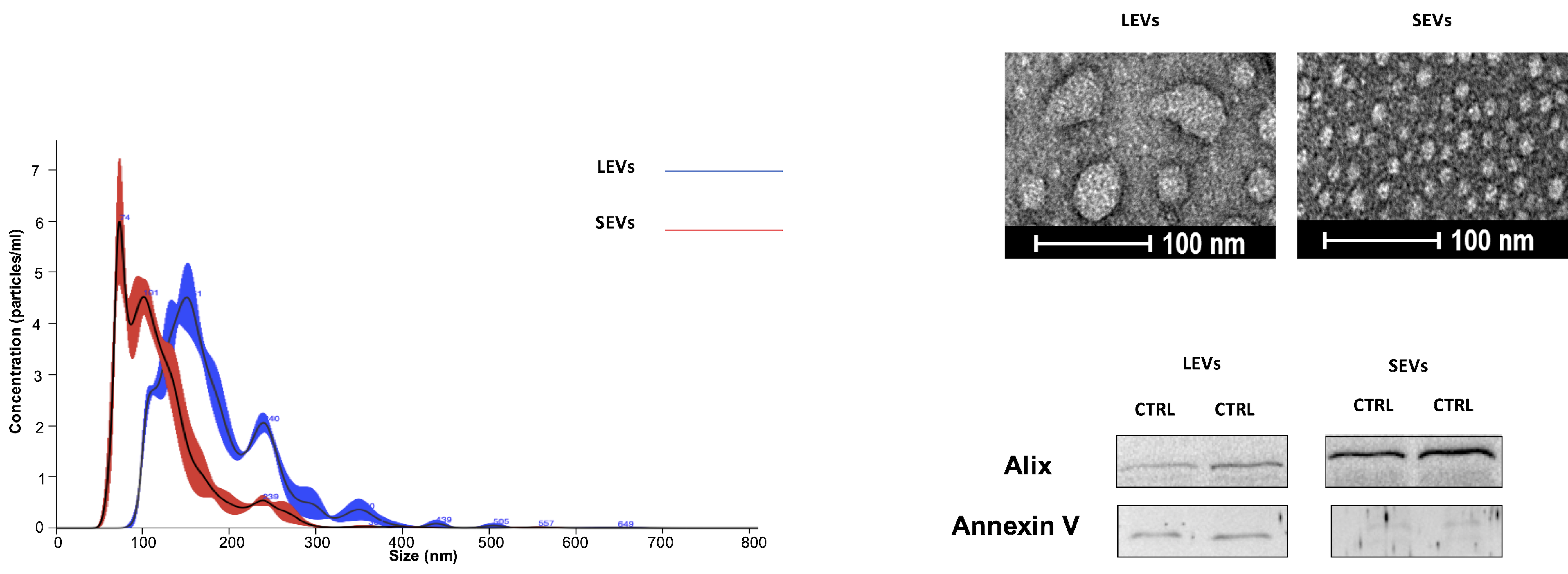

Supplement: Supplementary Figure 2 — Large extracellular vesicles (LEVs) and small extracellular vesicles (SEVs) characterization. (A) Nanosight profile of LEVs and SEVs from plasma of a control (CTR); (B) Representative images obtained by transmission electron microscopy (TEM) of LEVs and SEVs from plasma (Scale bar: 100 nm). (C) Western blot of LEVs and SEVs markers in LEVs and SEVs samples from two CTRs showed the presence of Annexin V only in the LEV pellet and more Alix presence in SEV fraction. [file Image_2.TIFF]

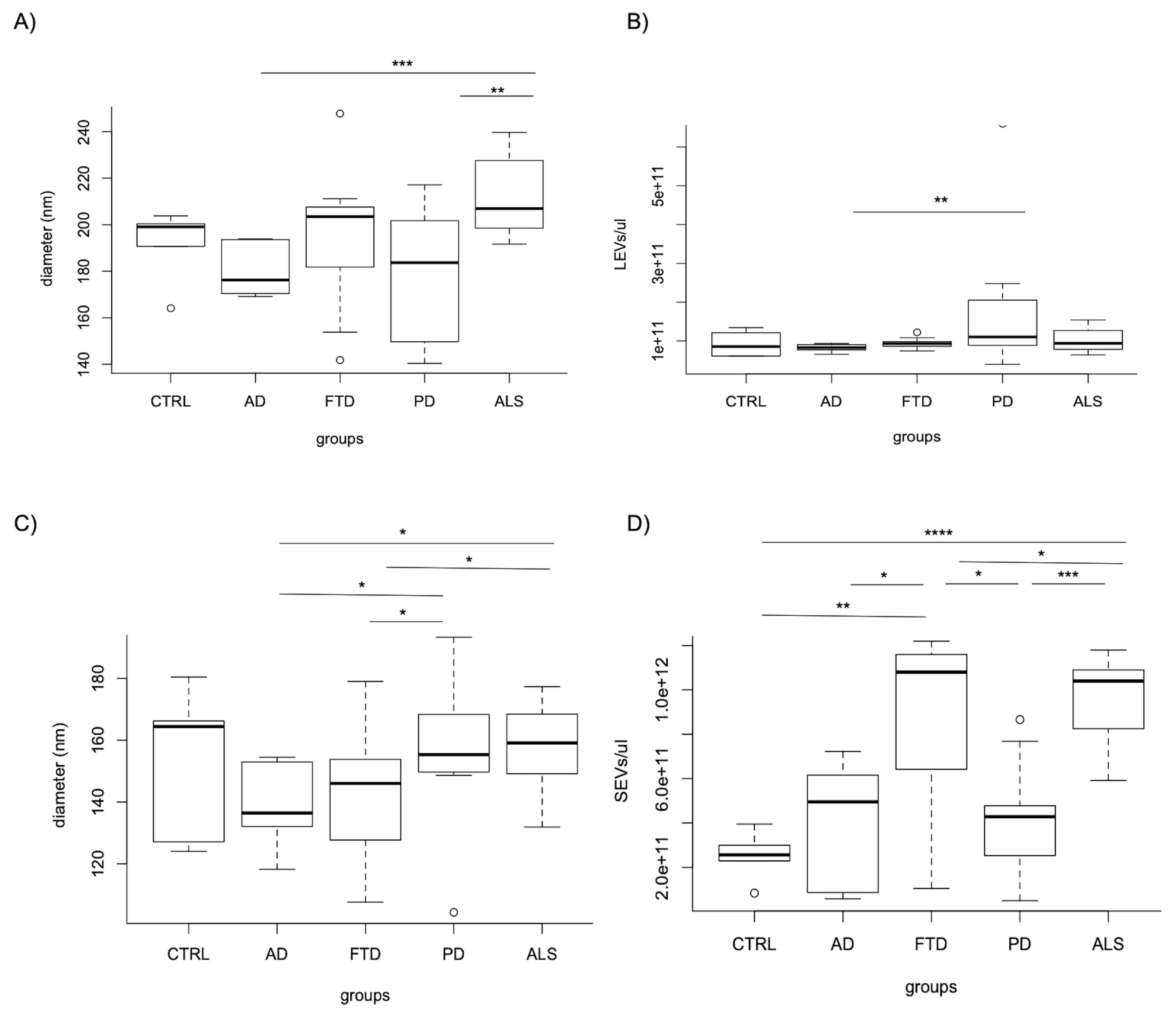

Supplement: Supplementary Figure 3 — Mean diameter and particle count concentration for LEVs and SEVs of the four NDs and CTRs. NTA was performed on LEVs and SEVs extracted from plasma of healthy controls and neurodegenerative diseases (NDs). The mean diameter size and count of LEVs (A,B) and SEVs (C,D) of CTRs and patients are reported in the boxplots (Dunn's test, *p < 0.05, **p < 0.01, ***p < 0.001, and ****p < 0.0001) (D). These data have to be confirmed with more patients and controls. [file Image_3.TIFF]
